# Supplementary material for: High-Throughput Screening of Antiviral Compounds Using a Recombinant Hepatitis B Virus and Identification of a Possible Infection Inhibitor, Skimmianine
Source: Viruses. 2024 Aug 22;16(8):1346. doi: 10.3390/v16081346 (PMC11360121; doi:10.3390/v16081346)
Supplement: Supplementary file 1 [file viruses-16-01346-s001.zip › viruses-3119005-supplementary/Figure S1.pptx]

## Slide 1
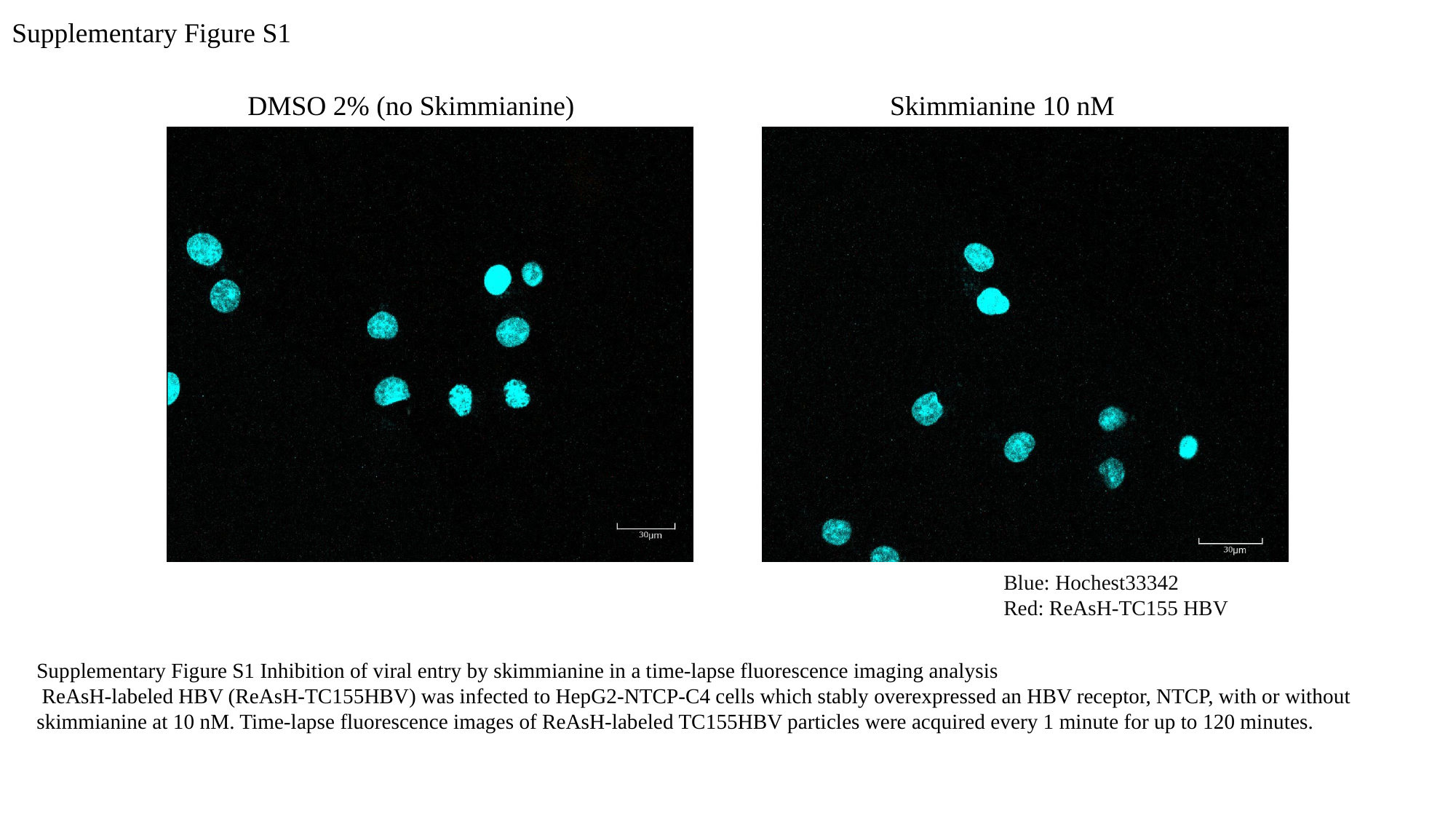

Supplementary Figure S1
DMSO 2% (no Skimmianine)
Skimmianine 10 nM
Blue: Hochest33342
Red: ReAsH-TC155 HBV
Supplementary Figure S1 Inhibition of viral entry by skimmianine in a time-lapse fluorescence imaging analysis
 ReAsH-labeled HBV (ReAsH-TC155HBV) was infected to HepG2-NTCP-C4 cells which stably overexpressed an HBV receptor, NTCP, with or without skimmianine at 10 nM. Time-lapse fluorescence images of ReAsH-labeled TC155HBV particles were acquired every 1 minute for up to 120 minutes.
